# Minimally Invasive Non-surgical Periodontal Therapy and Photoactivated Disinfection for the Treatment of Furcation Involvement

**DOI:** 10.7759/cureus.72193

**Published:** 2024-10-23

**Authors:** Marwan El Mobadder, Mahmoud El Kontar, Bachar Husseini

**Affiliations:** 1 Laser Laboratory, Oral Surgery Department, Wroclaw Medical University, Wroclaw, POL; 2 Oral Surgery and Implantology, Private Practice, Mount Lebanon, LBN; 3 Oral Surgery and Implantology, Dental Square Clinic, Beirut, LBN

**Keywords:** adult periodontitis, antimicrobial photodynamic therapy, diode laser therapy, furcation involvement, localized aggressive periodontitis, minimal invasion, non surgical treatment periodontitis, photoactivated disinfection, photodynamic therapy

## Abstract

Periodontitis is a biofilm-induced chronic inflammatory disease that, if left untreated, can result in alveolar bone and tooth loss. Intrabony defects and furcation involvement (FI) are particularly difficult to manage, as they often persist after step 1 and step 2 periodontal therapy.

In this case, we report a relatively novel therapeutic approach to managing deep furcation involvement in the first mandibular right molar (#46). The concerned area of furcation involvement presented a localized periodontitis with a Silness-Loe plaque index of 3, positive bleeding on probing, gingival recession of 2 mm, periodontal pocket depth (PPD) of 7 mm, and clinical attachment loss (CAL) of 9 mm. Moreover, the preoperative periapical X-ray showed a clear radiolucency between the roots of #46, indicating alveolar bone loss in the concerned area. The treatment consisted of step 1 and step 2 periodontal therapy according to the recommendations of the European Federation of Periodontology (EFP) and the American Academy of Periodontology (AAP). One week later, minimally invasive non-surgical periodontal treatment (MINST) and photoactivated disinfection (PAD) protocol were performed on the concerned FI. MINST was achieved with ultrasonic instrumentation using the TK2-1L and TK2-1R (Perio Maintenance, ultrasonic tips, Newtron, P5 XS Acteon, France). PAD was performed using tolonium chloride (TC) as a photosensitizer and a 635 nm semiconductor diode laser (Smart M laser, Lasotronix, Warsaw, Poland) as a light source. The irradiation parameters were set at 200 milliWatts (mW), applied for 30 seconds with an energy density of 4774.65 J/cm², using a 400-µm fiber diameter. Both contact and continuous modes were employed, with the protocol repeated three times during the same session.

After six months of follow-up, a significant reduction of PPD and CAL was observed with values of 3 mm and 5 mm, respectively. Evidence of healed bone between the roots was noted on the peri-apical X-ray. The treatment, which consisted of MINST and PAD, was considered successful due to the significant improvement in the clinical periodontal parameters, the evidence of bone reparation, and patient satisfaction. This case report highlights the importance of minimally invasive non-surgical approaches and photoactivated disinfection in the management of periodontitis.

## Introduction

Periodontitis is a chronic inflammatory condition caused by biofilm accumulation, which affects the periodontium and gradually results in the loss of alveolar bone [1]. The extent and pattern of bone loss around the tooth root can differ, with defects categorized based on the number of remaining bone walls. These defects are typically classified as one-wall, two-wall, or three-wall defects, with the prognosis declining as fewer bone walls remain. Intrabony defects often persist after the initial stages of periodontal treatment, as noted by the European Federation of Periodontology (EFP) and the American Academy of Periodontology (AAP). This is largely due to the challenges in eliminating subgingival biofilm [1].

In cases where bone loss reaches the furcation of multi-rooted teeth, furcation involvement (FI) may be identified. FI is classified into stages, ranging from Class I to Class III, with the severity increasing as the defect progresses to fully affect the furcation area. The presence of irregular bony architecture and unfavorable anatomies, such as intrabony defects and furcations, is indirectly linked to residual pockets [2, 3]. It is well known that sites with residual pockets ≥5 mm are at higher risk for further attachment loss and tooth loss. Due to the increased risk of disease progression associated with intrabony defects, surgical intervention is often required to prevent further deterioration of the dentition [2,3]. Clinical guidelines for treating Stage I-III periodontitis recommend regenerative or reconstructive surgery or the repetition of the non-surgical mechanical debridement for residual pockets associated with intrabony defects ≥3 mm deep [4]. However, research has shown that the use of biomaterials is not always necessary for regeneration [4]. Instead, optimizing the local environment to enhance the intrinsic healing potential of periodontal tissues is often recommended [4]. Consequently, there has been a shift towards more patient-friendly, minimally invasive techniques, a concept adapted from medicine [4].

In periodontology, minimally invasive non-surgical treatment (MINST) of periodontitis has been proposed as an alternative approach for treating intrabony defects, focusing on minimizing tissue trauma and enhancing wound healing by avoiding surgical incisions and sutures [5]. For instance, in a retrospective analysis of 35 intrabony defects treated with MINST, there was an overall mean reduction of 3 mm in radiographic intrabony defect depth. The average reduction in periodontal pocket depth (PPD) and clinical attachment loss (CAL) were 3.12 mm and 2.78 mm, respectively, with minimal increase in recession [5].

Another promising and minimally invasive non-surgical approach is photoactivated disinfection (PAD) protocol, also known as photodynamic therapy or activated photodynamic therapy [6]. PAD has shown promising results in the reduction of clinical periodontal parameters in patients with periodontitis. PAD is a minimally invasive, non-surgical technique that utilizes a photosensitizer or photosensitizing agent, which is activated by light from a laser or other light source. The interaction between the light and the photosensitizer leads to the selective destruction of targeted cells or bacteria. For instance, El Mobadder et al. demonstrated in a study that a statistically significant reduction in periodontal pocket depth (PPD) was observed after 3 months of follow-up in the scaling root planning (SRP)+PAD group compared to SRP alone with 3.79 mm for SRP and 4.85 mm for SRP+PAD, respectively [6]. The aim of this case report is to evaluate the effectiveness of combining PAD with the MINST procedure in managing a severe case of furcation involvement. To the best of our knowledge, this is the first case report that combines PAD and MINST in the management of furcation involvement.

## Case presentation

**Figure 1:**
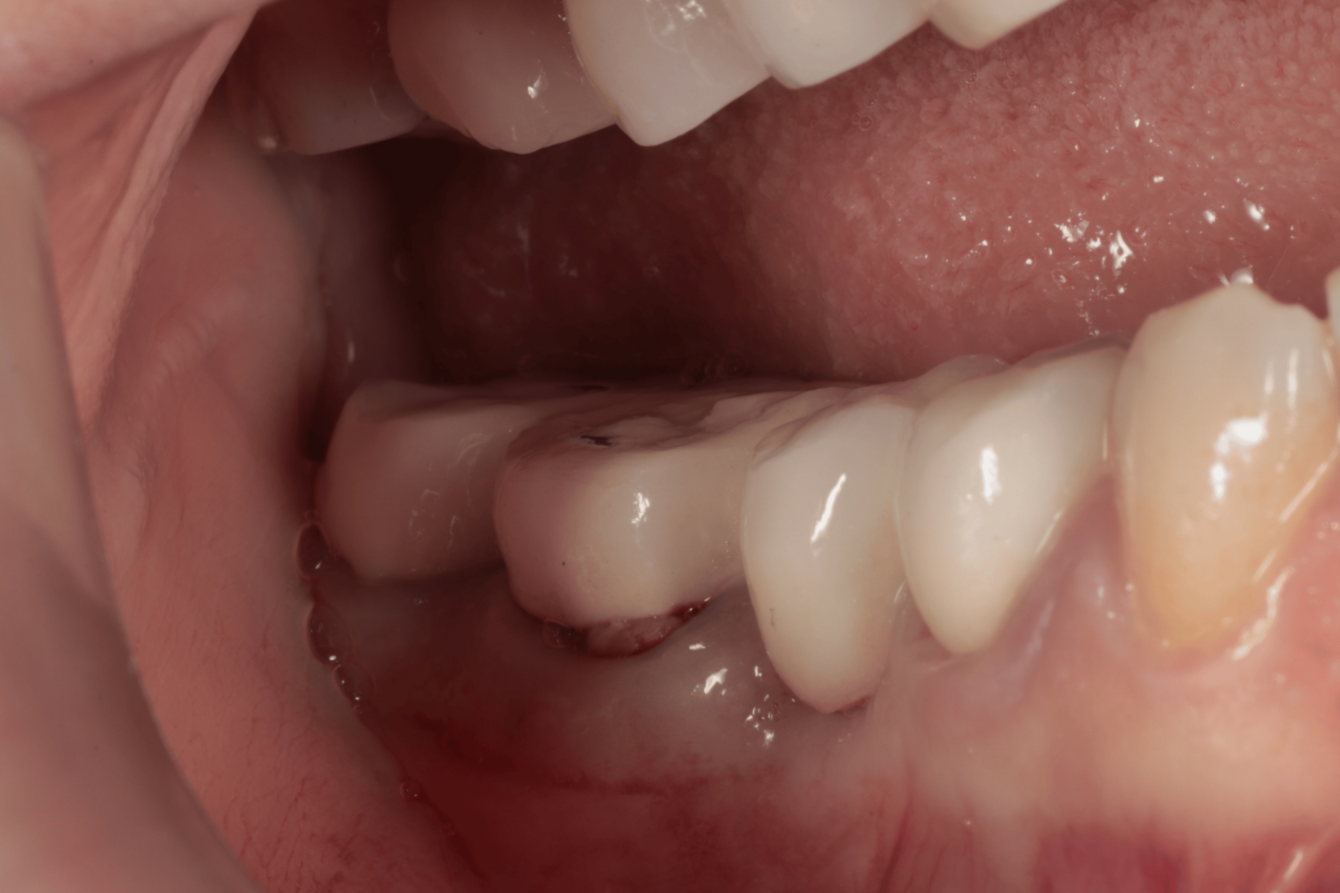
Clinical aspect of the first right mandibular molar preoperatively

**Figure 2:**
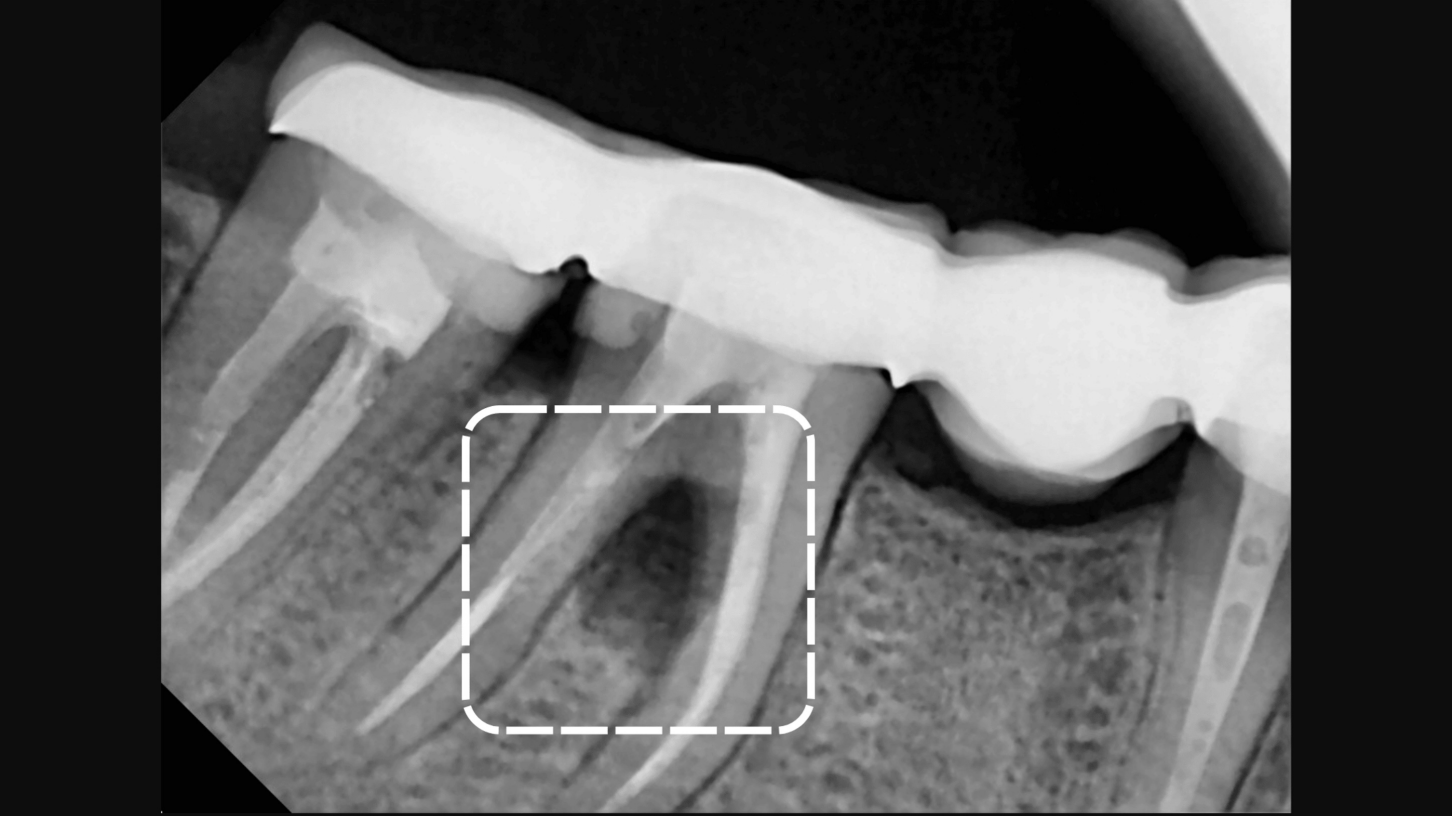
Radiographical aspect of the first right mandibular molar preoperatively

A 47-year-old caucasian female non-smoker presented to the clinic with a chief complaint of deep pain in the mandibular right arch. The patient had no systemic conditions known to affect her overall periodontal or dental health. Clinical and radiographic examinations revealed furcation involvement, along with a radiolucency observed on the periapical radiograph. The clinical periodontal parameters of the furcation involvement area (a buccal aspect of tooth 46) were as follows: a plaque index score of 3 according to the Silness-Löe classification, positive bleeding on probing, 2 mm of gingival recession, a periodontal pocket depth of 7 mm, and a clinical attachment loss of 9 mm. Additionally, a palpable lymph node was noted in the right mandibular arch in the same region, Figures 1, 2. During the first session, oral hygiene instructions were provided to the patient based on the recommendations of the EFP and AAP. The patient was prescribed an appropriate interdental brush and educated on its use. The treatment included step 1 and step 2 periodontal therapy, following the guidelines of the EFP and AAP, using both manual and ultrasonic instrumentation. Full-mouth scaling and root planing (SRP) were carried out in a single session. Following the SRP procedure, 0.12% chlorhexidine was applied into the sulcus for rinsing to aid in disinfection and promote healing.

Minimally invasive non-surgical treatment

**Figure 3:**
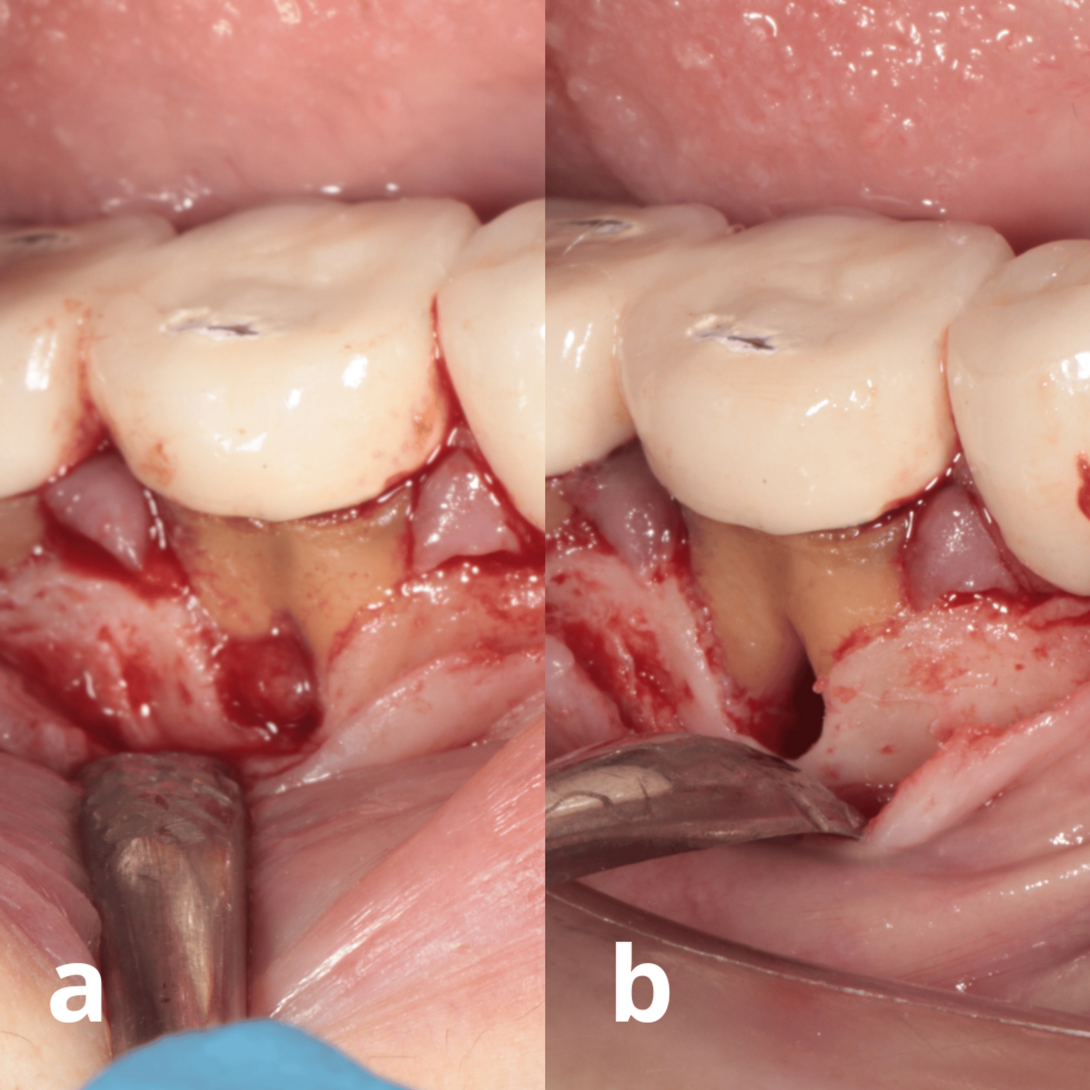
Clinical aspects during minimally invasive non-surgical periodontal therapy: a) After flap elevation, note the granulation tissue between the furcations. b) After mechanical debridement, observe the absence of granulation tissue between the roots.

The treatment protocol was carried out using 2.4X magnification loupes for enhanced visualization. Local anesthesia without adrenaline was administered to prevent vasoconstriction, which could negatively affect periodontal healing. A sub-papillary approach was employed to minimize trauma to the soft tissues, particularly the coronal portion of the interdental papilla. Under direct vision, thorough and gentle debridement of the root surface to the base of the pocket was performed. TK2-1L and TK2-1R dental tips (Perio Maintenance, ultrasonic tips, Newtron, P5 XS Acteon, France) were used. Care was taken to avoid smoothing the root surface or performing gingival curettage, Figure 3.

Photoactivated disinfection protocol 

**Figure 4:**
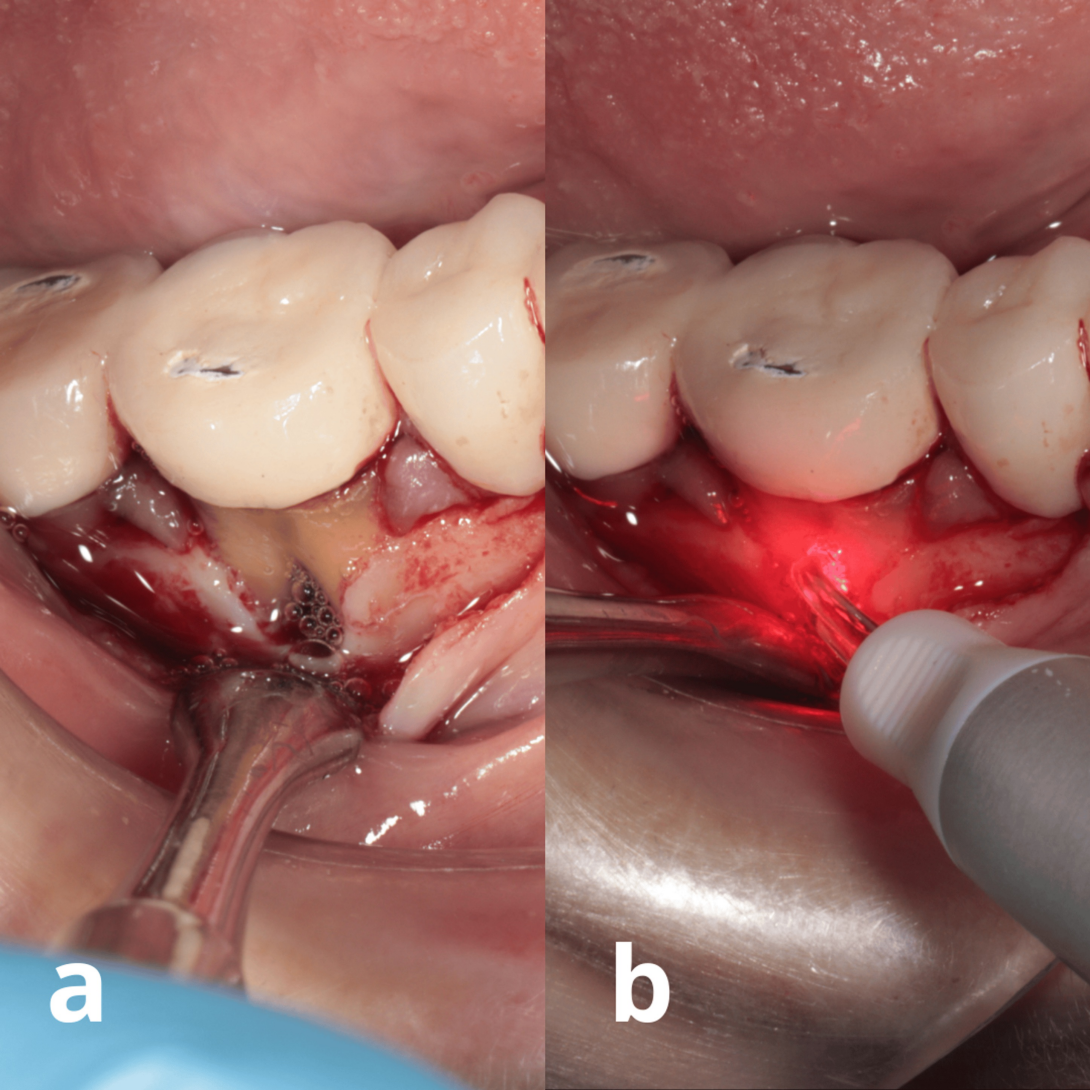
Clinical aspects during PAD: a) Note the presence of tolonium chloride between the roots b) During irradiation with the 635 nm wavelength of a diode laser (Smart M laser, Lasotronix, Warsaw, Poland)

PAD was conducted using a diode laser with a 635 nm wavelength (Smart M, Lasotronix, Warsaw, Poland) along with TC solution as the photosensitizing agent (PAD Smart Solution, Lasotronix). The irrigation process was performed slowly and paused once the TC solution filled the intrabony defect in the furcation. Afterward, a 60-second waiting period was observed. Then, a Perio Applicator (Lasotronix, Warsaw, Poland), designed for photoactivated disinfection protocol, was carefully inserted into the pocket at the appropriate depth, and laser irradiation was initiated. The irradiation parameters applied were 200 mW for 30 seconds, with an energy density of 4774.65 J/cm² and a fiber diameter of 400 µm, utilizing both contact and continuous modes of operation. The irradiation procedure was repeated two more times at the same furcation site during the treatment session, resulting in a total of three repetitions. During each irradiation, the tip was moved from the apical to the coronal direction in a steady manner, advancing slowly at approximately 1 mm per second to ensure complete coverage of the pocket of the intrabony defect. After PAD, the goal was to encourage the formation of a stable blood clot by allowing natural blood flow into the intrabony defect, and subgingival rinsing was avoided at the conclusion of the treatment to prevent disturbing the clot formation, Figure 4.

Follow-up

**Figure 5:**
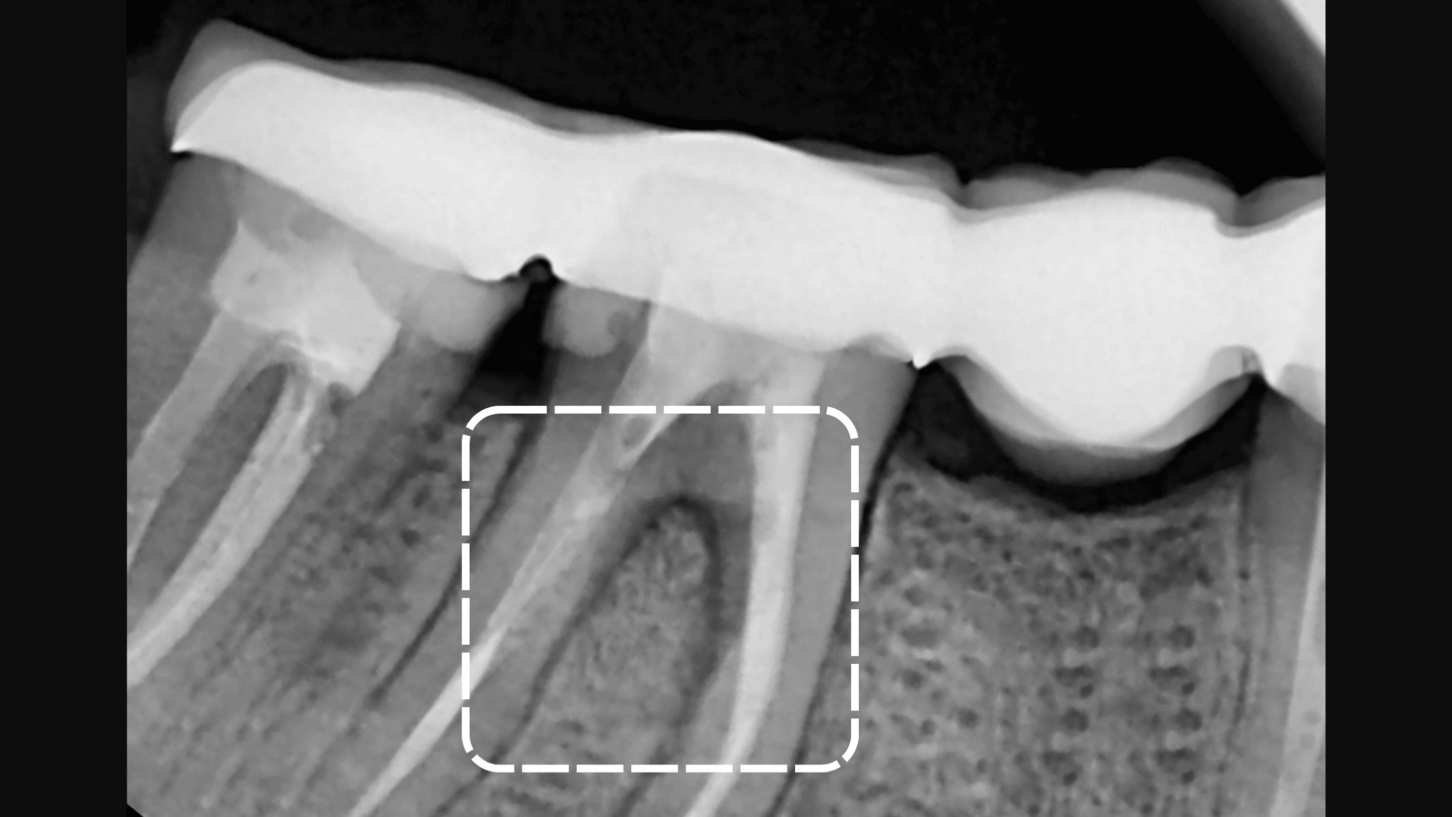
Periapical radiography of the first right mandibular molar at six months of follow-up.

Six months after treatment, the clinical periodontal parameters showed significant improvement. The Silness-Loe plaque index was recorded at 1, with no bleeding on probing for tooth #46. The periodontal pocket depth at the furcation (buccal aspect of #46) was reduced to 3 mm, gingival recession measured 2 mm, and the clinical attachment level in the furcation area was 5 mm, representing a marked reduction from the initial clinical attachment level of 9 mm. Furthermore, the palpable lymph node had disappeared. Radiographically, a periapical X-ray revealed evidence of bone repair in the area of furcation involvement, suggesting a resolution of the inflammation and successful periodontal regeneration. Overall, the patient expressed satisfaction with the treatment outcomes, Figure 5.

## Discussion

Furcation involvement and intrabony defects present a considerable challenge in periodontal treatment due to its complex anatomy and limited accessibility for mechanical debridement. The present case report demonstrates the successful application of MINST in combination with PAD for treating furcation involvement, providing an effective and minimally invasive alternative. This approach resulted in significant clinical improvements, including reduced probing depth (7 mm to 3 mm) and clinical attachment loss (9 mm to 5 mm) with no change in gingival recession (remaining 2 mm), along with bone regeneration and the resolution of infection.

In addition, the patient reported pain resolution and the disappearance of a palpable lymph node. While pain resolution may confirm a reduction in periodontal inflammation and infection, the disappearance of the palpable lymph node cannot be attributed to the treatment but is important to note. The use of MINST, in this case, aimed to minimize trauma to the periodontal tissues, particularly the interdental papilla, while ensuring effective removal of the biofilm [5]. The effectiveness of MINST was highlighted in a prospective cohort study by Mehta et al., which showed notable improvements in PPD and CAL after 12 months. In the study, 66.7% of defects achieved pocket closure (PPD ≤ 4 mm), and 58.3% met the composite outcome of PPD ≤ 4 mm with a CAL gain of ≥ 3 mm [7]. Deeper defects and narrower angles were associated with better radiographic and clinical outcomes, respectively [7]. As a result, MINST was selected as the preferred treatment in this case report. Alongside MINST, PAD was also used, involving a 635 nm diode laser and tolonium chloride as the photosensitizer.

The use of PAD has been increasingly recognized for its ability to effectively reduce subgingival bacterial load without the use of systemic antibiotics, which is particularly beneficial in the current context of antibiotic resistance. The laser energy, combined with the photosensitizer, results in the formation of reactive oxygen species that effectively target and destroy periodontal pathogens. For instance, PAD within a specific protocol has been proven to have strong bactericidal properties against *Porphyromonas gingivalis*, *Aggregatibacter actinomycetemcomitans,* and other pathogens of the red complex [8, 9, 10]. However, other studies have suggested that PAD does not provide a statistically significant improvement in the overall clinical periodontal parameters in patients with chronic periodontitis [11, 12]. Moreover, another issue with PAD is that the studies are heterogeneous in their choice of photosensitizer (PS), light source, and irradiation parameters [13]. This approach helped disinfect in depth the area of furcation involvement with a significant reduction of the total bacterial count and the count of the periodonto-pathogens; hence, resulting in a better repair of the periodontium. This was confirmed not only by the improvement of the clinical periodontal parameters but also by the evidence of bone repair seen in a six-month follow-up periapical radiograph.

Thus, in this case, the use of PAD helped to enhance the overall antimicrobial effect of the treatment, contributing to the significant clinical improvements observed. The success observed in this case aligns with the growing body of evidence supporting the concept of leveraging the body's intrinsic healing capacity through minimally invasive approaches while optimizing the local environment. However, it is important to address the limitations of this case report. The results presented are based on a single patient, and while they are promising, they cannot be generalized without further research involving larger sample sizes and randomized controlled trials.

## Conclusions

This case report highlights the potential of combining minimally invasive non-surgical periodontal therapy and photoactivated disinfection to effectively manage severe furcation involvement, which often persists after standard non-surgical treatment. This method reduces the need for surgical intervention, supports healing, and leads to favorable clinical results, as demonstrated by a substantial decrease in periodontal pocket depth, clinical attachment loss, and evidence of bone regeneration in the furcation area. Future research should focus on evaluating the long-term effectiveness of this combined approach, its applicability to a larger cohort, and comparisons with a control group.
